# Neurobiological Links between Stress, Brain Injury, and Disease

**DOI:** 10.1155/2022/8111022

**Published:** 2022-05-25

**Authors:** Hanmu Guo, Lexin Zheng, Heng Xu, Qiuyu Pang, Zhiyang Ren, Yuan Gao, Tao Wang

**Affiliations:** Department of Forensic Medicine, School of Basic Medicine & Biological Sciences, Soochow University, Suzhou 215123, China

## Abstract

Stress, which refers to a combination of physiological, neuroendocrine, behavioral, and emotional responses to novel or threatening stimuli, is essentially a defensive adaptation under physiological conditions. However, strong and long-lasting stress can lead to psychological and pathological damage. Growing evidence suggests that patients suffering from mild and moderate brain injuries and diseases often show severe neurological dysfunction and experience severe and persistent stressful events or environmental stimuli, whether in the acute, subacute, or recovery stage. Previous studies have shown that stress has a remarkable influence on key brain regions and brain diseases. The mechanisms through which stress affects the brain are diverse, including activation of endoplasmic reticulum stress (ERS), apoptosis, oxidative stress, and excitatory/inhibitory neuron imbalance, and may lead to behavioral and cognitive deficits. The impact of stress on brain diseases is complex and involves impediment of recovery, aggravation of cognitive impairment, and neurodegeneration. This review summarizes various stress models and their applications and then discusses the effects and mechanisms of stress on key brain regions—including the hippocampus, hypothalamus, amygdala, and prefrontal cortex—and in brain injuries and diseases—including Alzheimer's disease, stroke, traumatic brain injury, and epilepsy. Lastly, this review highlights psychological interventions and potential therapeutic targets for patients with brain injuries and diseases who experience severe and persistent stressful events.

## 1. Introduction

Stress [1] refers to a systemic nonspecific response that occurs when the body is stimulated by various environmental factors or social and psychological factors. The brain is an important organ in stress and its adaptive regulation, ultimately determining the outcome of stress through neuroendocrine, immune, and metabolic mechanisms [2]. It has been found that mild and moderate stress can cause the body to regulate homeostasis and minimize the potential impact of the threat, which is of important defensive significance for the brain against injuries and diseases. However, intense and persistent stress [3] can trigger a variety of psychological and cognitive deficits by causing or aggravating injuries and diseases involving key brain regions such as the hippocampus, amygdala, hypothalamus, and prefrontal cortex (PFC). The mechanisms are closely linked [4] to excessive releases of various stress hormones, neurotransmitters, and neurotrophic factors caused by overactivation of the hypothalamus-pituitary-adrenal (HPA) axis and the locus coeruleus/norepinephrine (LC/NE) system. In addition, activation of endoplasmic reticulum stress (ERS), apoptosis, oxidative stress, and excitatory/inhibitory neuron imbalance are also important mechanisms for stress to affect normal brain regions and disease prognosis [5, 6]. Not only does stress damage the structure and function of normal brain regions [7, 8], studies have shown that it can lead to cognitive impairment, anxiety, and depression and aggravate neurological dysfunction caused by brain injuries and diseases such as Alzheimer's disease (AD), stroke, traumatic brain injury (TBI), and epilepsy [9, 10].

This article reviews research progress regarding the effects and mechanisms of stress on key brain areas and the neurobiological links between stress and brain injuries and diseases. This review is aimed at highlighting potential psychological interventions and therapeutic targets for patients with brain injuries and diseases who experience severe and persistent stressful events.

## 2. Stress Models and Their Significance

According to the duration of the stressor, stress can be divided into acute and chronic subtypes. Due to different experimental needs, various stress models have been developed using diverse stressors [11], including hot and cold stimulation, electric foot shock, restraint stress (RS), predator-based psychosocial stress (PPS), social failure, chronic unpredictable mild stress (CUMS), social isolation, time-dependent sensitization (TDS), underwater trauma (UT), and single persistent stress (SPS). These different stress models have special significance for simulating diseases or pre- and postdisease conditions. The following section describes several classic and common stress models (Table 1).

Electric foot shock is a complex stressor comprising physiological and emotional components. Foot shock has been used as an important tool in studies involving animal models in the field of psychopharmacology [12]. The experimental paradigm involves acute or chronic exposure to shocks of different intensities and durations on the floor of a live grid in an electric foot shock device. Research [12, 13] has shown that different intensities of foot shock can produce behavioral and neurochemical changes reflecting human depression, anxiety, and posttraumatic stress disorder (PTSD). This model has the experimental advantages of being able to control the intensity and duration of the stressor. The “learned helplessness” induced by electric shock simulates the symptomatology of depression and is used to establish an animal depression model [12].

The RS model involves fixing an animal to a wooden board or using a special restraint syringe [14]. Chronic restraint stress (CRS) can lead to behavioral inhibition, anxiety, and depression-like behaviors [14], downregulate the expression of glucocorticoid receptors (GR), and weaken the release of glutamate induced by brain-derived neurotrophic factor (BDNF) in the PFC [15]. Given the roles of the PFC in the pathology of depression and regulating emotional behavior and cognitive function, CRS is useful for the development of animal models of anxiety and depression [16, 17].

PPS involves exposing rodents to predators or predator odors, resulting in life-threatening experiences [18]. Following PPS, animals show high anxiety, impaired learning and memory, strong memory of trauma, excessive shock, increased central nervous system (CNS) and peripheral blood proinflammatory cytokines such as IL-1*β* and NLRP3 [19], and decreased serotonin levels and increased norepinephrine levels in the damaged hippocampus and PFC caused by PPS. Thus, PPS is a good model for studying the mechanisms, risk factors, and clinical treatment of PTSD [20].

The social failure model uses social conflict between members of the same species to produce emotional and psychological stress [21]. In an example experiment, rodents are exposed to different resident invaders for several days and the intruders are quickly investigated, attacked, and defeated by the residents [22]. After a few minutes of physical interaction, the residents and intruders are usually separated by a perforated plastic partition, which allows visual, olfactory, and auditory contact for the remaining 24 hours. The social failure stress paradigm is the most commonly used model for male rodents and is one of the most extensive animal models of depression in research [22].

The CUMS model is one of the most widely validated and realistic models of depression and shows satisfactory reliability [7]. In the CUMS paradigm, animals are exposed to a series of mild stressors (isolated or crowded housing, food or water shortage, interruption of dark/light cycle, inclination of their cage, wet padding, etc.) in an unpredictable way for several weeks or even months [8]. Following the CUMS procedure, rats show decreased sucrose intake, decreased expression of GR, and reduced release of glutamate induced by BDNF in the PFC [15]. According to the intensity and duration of CUMS, animals show depressive behaviors similar to human mental disorders, such as decreased pleasure, agitation, mental retardation, helplessness, sleep disorders, and reduced social communication. The repetition of the CUMS stressors is comparable to repeated stressors in human life and the natural conditions of major depressive disorder (MDD).

Early life stress (ELS) can result in behavioral and cognitive impairment in adults. The most commonly used rodent model of ELS is maternal separation (MS). Separating rodent pups from their mother and nest for 3 hours or longer per day during the first 2 weeks of life leads to increased anxiety-like behavior and cognitive impairments in adulthood and overactivation of the HPA axis to stress [23]. Behavioral and memory deficits and depressive symptoms are also associated with MS [24]. ELS has been shown to have a significant effect on DNA methylation in the hypothalamus in early life and lead to stress-specific attention deficit hyperactivity disorder (ADHD) in young adults [25]. The social and individual behaviors of adult mice can also be influenced by sex-specific early life events. Interestingly, MS does not change anxiety or social behavior in male mice but leads to increased anxiety and social behavior deficits in female mice. However, mild ELS may have a positive impact on later emotional behavior [23]. Short separation between pups and their mother leads to increased social behavior in both sexes and reduced anxiety in males [23]. MS is also used to study the link between stress and oxidative stress in the brain [26, 27], and it has been shown that alterations of brain lipid peroxidation and the antioxidant enzymes activities are age and sex dependent [28]. Under the termination of stress-hyporesponsive period (SHRP), MS reduced antioxidant enzyme activities and increased lipid peroxidation in male rats [28], while these parameters are stable in female rats.

Social isolation is also an ELS model [29]. In a typical experimental paradigm, animals are socially isolated for several weeks. Social isolation models are often used to simulate anxiety and depression, although attention must be paid to gender differences [30]. In animal models, social isolation has effects on glucocorticoids, corticosterone (CORT), adrenocorticotropic hormone (ACTH), glutamate, *γ*-aminobutyric acid (GABA), and other neurotransmitters [31]. Repeated unknowable stress combined with social isolation leads to weakening of the HPA axis response and persistent abnormal fear memories [32]. Meaningly, brain oxidative stress is a crucial contributor to the development of neuropathological alterations induced by social isolation [26]. Activity of copper, zinc-superoxide dismutase (CuZnSOD), and catalase in the hippocampus of rats decreased after acute social isolation [33]; lipid peroxidation in both the striatum and cortex also increased [34].

By studying different types of stress patterns, we can simulate various neuropsychiatric injuries and diseases. These patterns are thus conducive to understanding the pathogenesis and potential interventions for related neuropsychiatric diseases. The occurrence of neuropsychiatric diseases is not only related to the type and intensity of stress but also to functional and structural damage of different brain regions. The next section reviews the roles and underlying mechanisms of stress on key brain regions.

## 3. Neurological Links between Stress and Key Brain Regions

**Figure 1:**
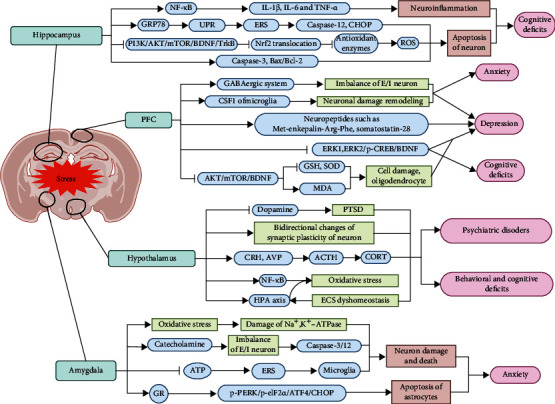
Effect of stress on key brain regions. Stress induces NF-*κ*B-mediated neuroinflammation, triggers endoplasmic reticulum stress (ERS), downregulates antioxidant enzymes and causes oxidative stress, or directly increases caspaes-3 and the Bax/Bcl-2 ratio to promote the apoptosis of hippocampal neurons, and finally leads to cognitive deficits. Stress causes anxiety and depression by leading to excitatory/inhibitory (E/I) neuron imbalance, increasing MDA, inhibiting GSH and SOD, destroying neuronal damage remodeling, and inhibiting the ERK-CREB-BDNF pathway in the prefrontal cortex (PFC). In the hypothalamus, stress induces behavioral and cognitive deficits and psychiatric disorders by activating the hypothalamus-pituitary-adrenal (HPA) axis, oxidative stress, and corticotropin-releasing hormone (CRH) neuron or leading to the bidirectional changes of synaptic plasticity of neuron. In the amygdala, stress makes neuron damage by leading to the imbalance of E/I neuron, damaging of Na^+^, K^+^-ATPase caused by oxidative stress, and activating p-PERK/p-eIF2*α*/ATF4/CHOP pathway-mediated astrocyte apoptosis, finally leading to anxiety. Note: UPR: unfolded protein response; GABA: glutamate, *γ*-aminobutyric acid; BDNF: brain-derived neurotrophic factor; GSH: glutathione; SOD: superoxide dismutase; MDA: malondialdehyde; PTSD: posttraumatic stress disorder; AVP: arginine vasopressin; ACTH: adrenocorticotropic hormone; CORT: corticosterone; ECS: endocannabinoid system; GR: glucocorticoid receptor.

Stress has significant effects on different regions of the brain [3], including the hippocampus, hypothalamus, amygdala, and PFC. Depression, anxiety, cognitive deficits, and even mental diseases caused by stress are closely related to functional and structural damage of the related brain regions. Thus, the neurobiological links between stress and these key brain regions are reviewed in this section in order to analyze the mechanisms of stress-related diseases (Table 2 and Figure 1).

### 3.1. Stress and the Hippocampus

The hippocampus plays an important role in learning, memory, stress, and emotion regulation [35]. The hippocampus shows high sensitivity and responsiveness to stress [36], and its structure and function can be affected by stress. In animal models, chronic stress may significantly reduce hippocampal volume [37–39]. Notably, clinical studies [40] have shown that depression patients have a smaller hippocampal size relative to controls. One important reason for the decrease in hippocampal volume induced by stress is the loss of neurons. However, the mechanism of hippocampal neuron loss and cognitive dysfunction caused by stress is very complex and requires further research [41].

Previous studies have demonstrated oxidative stress was involved in stress-induced hippocampal damage. Chronic mild stress (CMS) increased the protein peroxidation, lipid peroxidation, and catalase activity and decreased superoxide dismutase (SOD) activity in the hippocampus [6, 42]. Chronic cold stress could induce hippocampal apoptosis with drastically increased serum reactive oxygen species (ROS) level, as well as brain lipid peroxidation levels [42]. The above mechanism may relate to HPA axis activation caused by stress [43], because glucocorticoids can increase NADPH oxidase- (NOX-) dependent ROS in hippocampus neurons [44].

During early or mild stress model, BDNF and peroxiredoxin-1 in the hippocampus can be upregulated to play an antioxidant cytoprotective role [45, 46], because BDNF controls nuclear translocation of the master redox-sensitive transcription factor-nuclear factor erythroid 2-related factor 2 (Nrf2), which activates antioxidant defenses [47]. BDNF levels in the hippocampus were found to decrease when faced with severe or sustained stress [48–51], which may be involved in inhibiting phosphatidylinositol 3-kinase (PI3K)/AKT/mammalian target of rapamycin- (mTOR-) mediated BDNF/tropomyosin-related kinase B (TrkB) pathway [52]. Under stress, low level of BDNF may prevent Nrf2 translocation and consequently inhibit the activation of detoxifying/antioxidant enzymes, ultimately resulting in the generation of sustained oxidative stress [47]. High levels of oxidative stress may cause inflammation by activating nuclear factor-*κ*B (NF-*κ*B) [46, 53] and increasing inflammatory factors such as IL-1*β*, IL-6, and tumor necrosis factor-*α* (TNF-*α*) in stressed rats [52]. Oxidative stress eventually leads to the depression of animals after stress [47, 54, 55], and the symptoms of depression were significantly improved after using drugs that can increase the level of BDNF or show antioxidant effect in animals [47, 51, 52, 54]. Inhibition of fatty acid amide hydrolase (FAAH) can also be antidepressant through promoting neural progenitor proliferation and BDNF expression and reducing adrenal gland weight and oxidative stress in mice under the CUMS model [49].

The endoplasmic reticulum (ER) is the main structure promoting protein maturation and has the functions of storing Ca^2+^ and synthesizing lipids [56]. The ER is easily disturbed by stress, which leads to accumulation of unfolded and/or misfolded proteins in the ER. These accumulated proteins form three major ER transmembrane proteins, namely, double-stranded RNA-activated protein kinase-like ER kinase (PERK), inositol-requiring enzyme 1 (IRE1), and activating transcription factor 6 (ATF6), which are cleaved to produce glucose-regulated protein 78 (GRP78), which initiates the unfolded protein response (UPR) [57], ultimately causing ER stress (ERS). Prior studies [58] have shown that mild and moderate ERS can alleviate cell damage, whereas persistent and severe ERS can lead to cell death via apoptosis. Moreover, many studies have shown that ERS is closely related to neuronal cell death in neurodegenerative diseases [58]. Zhang et al. [3] found that CRS can increase the ratio of apoptotic cells and upregulate the activity of caspase-3 and the ratio of Bax/Bcl-2 in the hippocampus of rats. Spatial memory deficits in the CRS group were also more severe than those in the control group [3]. Salubrinal, which is an ERS inhibitor, has been shown to effectively reduce the apoptotic rate and improve cognitive impairment in rats after CRS [3]. Furthermore, chronic stress [59] can lead to hippocampal CA3 apical dendritic atrophy, which is linked to impaired spatial memory.

Taken together, these studies suggest that stress promotes apoptosis of hippocampal neurons by activating ERS and increasing oxidative stress level, ultimately leading to cognitive or affective impairment. Thus, reducing ERS and oxidative stress may be an effective prevention or treatment strategy for cognitive or affective impairment caused by stress.

### 3.2. Stress and the Hypothalamus

The hypothalamus is the structural and functional basis of the neuroendocrine system [43], and stress responses in vertebrates are closely related to the HPA axis. Activation of the HPA axis [60, 61] triggers neurons in the paraventricular nucleus (PVN) of the hypothalamus to release corticotropin-releasing hormone (CRH) and arginine vasopressin (AVP) and then stimulates the anterior pituitary gland to produce and secrete ACTH, which promote the release of glucocorticoids and adrenal androgens from the adrenal cortex into the blood circulation. Many studies have shown that continuous activation of the HPA axis can lead to physiological impairments and behavioral dysfunction [62, 63]. CRH neurons in the PVN are not only involved in activation of the HPA axis; they also play a vital role in mediating stress-related functions [64]. In addition to the above classical pathways, stress can also have a significant effect on the hypothalamus through regulating the oxidative stress pathway [65].

PPS can decrease dopamine levels in the rat hypothalamus [66], which may play a role in the pathophysiology of PTSD. Interestingly, stress of different durations can lead to bidirectional changes in the synaptic plasticity of neuroendocrine cells in the PVN of the hypothalamus [67]. After acute stress, presynaptic activity bursts and glutamatergic synapses change to multivesicular release patterns [67]. GABAergic synapses are conditionally excited after stress due to the collapse of the transmembrane chloride gradient and show inhibition of varying durations after stress [67]. Endorphin signals are highly unstable in the PVN [67] and are enhanced under acute stress, collapse under repeated stress, and are reset by new experiences after RS. It should be noted that activation of the HPA axis due to chronic stress exposure or repeated CORT treatment can lead to dyshomeostasis of the endocannabinoid system (ECS) in the hypothalamus, which may be involved in stress-related dysfunctions [43].

Both acute and chronic RS produced a significant increase in neuronal nitric oxide synthase (nNOS) mRNA in the PVN, medial parvocellular part, dorsolateral periaqueductal grey (DLPAG) in male Wistar rats [65], which depends on activation of NF-*κ*B [68], may cause accumulation of membrane aldehydic products, the disruption of blood-brain barrier (BBB), and the mitochondrial impairment [69, 70]. In previous studies, CORT increased oxidative stress [71]. Thus, oxidative stress can be reduced by inhibiting the HPA axis or blocking CORT release [72], recovering the depleted GSH and its enzymes, SOD activity [73], and participating in the antidepressant process [51]. This suggests that stress may directly promote oxidative stress and cause damage, or it may cause oxidative stress and then cause damage by activating the HPA axis.

In conclusion, stress can alter the plasticity of the hypothalamus and directly affect neuronal activity, leading to behavioral and cognitive deficits and even psychiatric disorders. Stress may also affect other hypothalamic hormones such as dopamine and the ECS via the HPA axis [43], although the specific mechanisms require further research. Oxidative stress caused by stress can damage the hypothalamus; however, the relationship between oxidative stress and the HPA axis needs further clarification.

### 3.3. Stress and the Amygdala

The amygdala is one of the key brain regions associated with the stress response and stress-induced mental conditions such as anxiety [74]. The amygdala [75] is a structurally complex brain region that is divided into the basolateral amygdala (BLA), medial nucleus of the amygdala (MEA), and central nucleus of the amygdala (CEA). Repeated social isolation stress [4] has been shown to induce dendritic branching in BLA pyramidal neurons in rats, which is accompanied by increased social avoidance behavior, indicating that stress induces social anxiety. Stress [76] can also promote apoptosis of amygdala astrocytes and upregulate the expression of GR, CHOP, and caspase-12. According to an *in vitro* study, dexamethasone (DEX) can induce apoptosis of human astrocytes and inhibition of the ERS-related protein CHOP pathway can alleviate DEX-induced apoptosis [77]. The above study suggests that CORT may be involved in stress-induced anxiety by activating p-PERK/p-eIF2*α*/ATF4/CHOP pathway-mediated apoptosis.

Stress can lead to neurodegeneration and neuronal death in the BLA of rats [78], which may be related to increased serum catecholamine and imbalance of excitatory/inhibitory (E/I) neurons in the amygdala. Thionine and Fluoro-jade B staining [78] has revealed that, with extension of stress duration, the amount of degeneration and necrosis of neurons in the BLA increases. Therefore, ERS-mediated GABAergic neuronal injury in the BLA may be an important cause of stress-induced mental disorders [79]. Stress can result in a continuous decrease of ATP in the amygdala, thereby affecting the energy supply of neurons [78]. Such a decrease in ATP in the amygdala can cause neuron damage by inducing ERS and the overactivation of microglia [78]. Furthermore, hyperpermeability and damage of the BBB induced by RS in the amygdala are associated with behavioral and cognitive dysfunctions and neurodegenerative disorders [78].

Stress can also damage the amygdala through activating oxidative stress. Oxidative stress caused by chronic stress in the amygdala can lead to memory defects and inhibitory avoidance, which may relate to damage in lipids and (or) Na^+^, K^+^-ATPase structure and activity [80], because membrane forming and signaling lipids in the brain closely related to the etiopathologies of depression, bipolar disorders, and schizophrenia [81].

In summary, activation of ERS, oxidative stress, and BBB damage appear to play crucial roles between stress and the amygdala.

### 3.4. Stress and the PFC

The PFC is responsible for various cognitive functions such as working memory, planning, decision-making, error monitoring, and emotion regulation by regulating the balance between E/I neurotransmissions [82]. Impairment of cognitive flexibility is mainly associated with the monoamine system of the PFC [82]. Oxidative stress is also a crucial way of stress to affect the PFC [55, 83]. As depression often cooccurs with cognitive impairment, the PFC is considered an important brain region in depression [82].

During the development of the PFC, normal E/I balance of neurons is necessary for the maturation of PFC-dependent functions [82], including emotion regulation. Long-term stress in adulthood can affect E/I balance in the PFC and thus lead to emotional disorders such as depression and anxiety [84]. Excessive inhibition of the PFC caused by increased GABAergic system activity [85] can lead to affective disorders [86]. In contrast, stimulation of the PFC can reduce anxiety and depression-related behaviors in rodents [87] and eliminate learned fear [88]. It should be noted that, after chronic stress, dendritic retraction was observed in the PFC of male but not female rodents; some females even showed dendritic hypertrophy [89], suggesting that females may be more vulnerable to new stressors. The plasticity of the PFC thus appears very important for stress recovery and chronic or repeated stress may impair PFC plasticity—especially in women.

The molecular mechanisms through which stress affects the PFC have been widely studied. For example, the anxiety and depression-like behaviors induced by CUMS may be related to increased colony-stimulating factor (CSF) 1 in microglia and neuronal synaptic defects in the medial PFC (mPFC) [90].

Stress can also downregulate neuropeptides like Met-Enkephalin-Arg-Phe, Met-Enkephalin-Arg-Gly-Leu, peptide PHI-27, somatostatin-28 (AA1-12), and little SAAS in the PFC; as the regulation of neuropeptides is brain region specific, it may be related to depression-related behaviors [91]. In social failure stress model rats, ERK signaling molecules, including pERK1 and pERK2 and the downstream molecules pCREB and BDNF, were significantly decreased in the lateral orbitofrontal cortex (OFC) and mPFC compared to the control group. Thus, ERK-CREB-BDNF signaling in the mPFC may be a common mechanism of emotional and cognitive changes in depression [92].

Moreover, after stress, rats exhibited a significant increase of the content of protein carbonyl, total glutathione (GSH) [55, 83], and malondialdehyde (MDA) level and a decrease of SOD activity or BDNF level in PFC [50, 83, 93]. Oxidative stress induced by chronic stress can lead to mPFC damage and decreased hippocampal neurogenesis [94], eventually leading to depression [47, 51, 55, 83, 93, 94] or anxiety-like behavior [27, 50]. CUMS exposure also significantly decreased GSH, BDNF, phosphorylation of AKT, and mTOR in PFC [50, 51, 93], and antioxidant drugs such as resveratrol [93], 5-HT [95], or carvedilol [96] can increase BDNF, AKT, and mTOR and decrease oxidative stress, significantly alleviating depression. Stress-induced depression may be also related to oligodendrocyte function in the PFC [97, 98]; the increase of oligodendrocytes in PFC can be observed after chronic stress [50], which may be body compensation [99]. Antidepressant escitalopram works through antioxidation [100–102]; it can inhibit lipid peroxidation (MDA) and increase antioxidant molecules (GSH, SOD) and BDNF in the PFC. Escitalopram restored the number of compensatory oligodendrocytes in the PFC [50], which means that the compensatory increase or decrease of oligodendrocyte function caused by chronic stress may be related to oxidative stress, but its exact mechanism needs to be further studied.

In conclusion, the brain shows strong regulation and adaptability to stress. Stress leads to neurological deficits by regulating the E/I balance of neurons and excessive oxidative stress in key brain regions. In future research, it will be possible to accurately study the neurological links between different brain regions or nuclei during the state of stress using optogenetics, single-cell sequencing, and other cutting-edge techniques.

## 4. Neurobiological Links between Stress and Brain Injuries/Diseases

**Figure 2:**
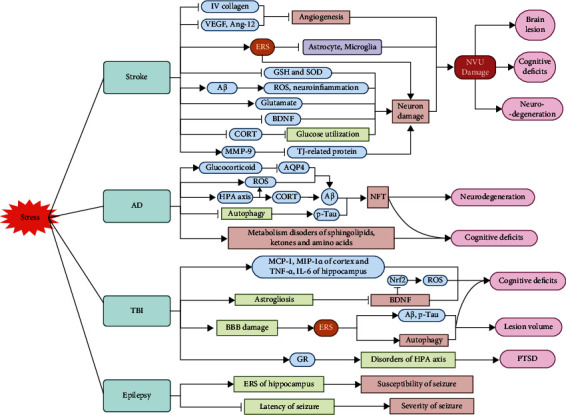
Impact of stress on brain diseases and injuries. In Stress+Stroke models, stress inhibits prognosis of stroke by restraining angiogenesis, inhibiting GSH and SOD, triggering endoplasmic reticulum stress (ERS) and aggravating neuron damage, and then hindering the recovery of neurovascular unit (NVU), finally resulting in larger brain lesions, more severe cognitive impairments, and neurodegeneration. In Stress+Alzheimer's disease (AD) models, stress promotes accumulation of nerve fiber tangles (NFT) by inhibiting AQP4, activating oxidative stress, making hypothalamus-pituitary-adrenal (HPA) axis overactivation, and inhibiting autophagy, which finally causes neurodegeneration and aggravates cognitive deficits with metabolism disorders of sphingolipids, etc. In Stress+traumatic brain injury (TBI) models, stress aggravates cognitive deficits by increasing monocyte chemoattractant protein-1 (MCP-1), macrophage inflammatory protein 1*α* (MIP-1*α*), tumor necrosis factor-*α* (TNF*α*), ROS, and interleukin-6 (IL-6), inhibiting brain-derived neurotrophic factor (BDNF), Nrf2, and damaging blood-brain barrier (BBB) and causes posttraumatic stress disorder (PTSD) by making HPA axis disorders. In Stress+Epilepsy models, stress raises susceptibility of seizure by activating ERS of the hippocampus and aggravates seizure by shortening the latency of seizure. Note: VEGF: vascular endothelial growth factor; ROS: reactive oxygen species; GSH: glutathione; SOD: superoxide dismutase; Nrf2: nuclear factor erythroid 2-related factor 2; CORT: corticosterone; MMP-9: metalloprotein-9; TJ: tight junction; GR: glucocorticoid receptor.

Stress leads to neurological deficits by damaging key brain regions. The pathophysiological processes of brain injuries and diseases, such as AD, stroke, TBI, and epilepsy, are linked to the related brain regions through various mechanisms including the neuroendocrine system, neuroinflammation, oxidative stress, and ERS [103, 104]. Patients with brain injuries and diseases often suffer from various stressors that may aggravate their neurological impairments. Thus, attention should be paid to the roles and mechanisms of stress in the progression of brain injuries and diseases in order to effectively and accurately identify targets for intervention and treatment for such patients (Table 3 and Figure 2).

### 4.1. Stress and AD

AD neuropathology is characterized by overproduction of A*β*, which deposits amyloid plaques that become insoluble and then aggregates and forms nerve fiber tangles (NFT) by accumulating p-Tau protein [105]. In studies of the pathogenesis of AD, much attention has been paid to the removal of A*β* [106]; the abnormal increase of A*β* has also attracted attention. A*β* can be removed from the brain by various scavenging mechanisms; the most important of which is BBB transport [107]. Recent studies have shown that the acetylcholine (Ach) system may play an important role in the removal of A*β* [108]. In the CNS, the Ach system, as part of the lymphatic system, rapidly removes waste from the brain interstitium [109], which is thought to be mediated by aquaporin 4 (AQP4). Wei et al. [110] reported that CUMS can damage the brain lymphatic system via the glucocorticoid signaling pathway to significantly reduce the expression of AQP4 and increase the accumulation of A*β*42 in the cerebral cortex; in contrast, a GR antagonist was found to reverse these effects [110].

As mentioned earlier [60, 61], stress activates the HPA axis and leads to increased plasma levels of ACTH and CORT. The HPA axis functions (e.g., chronic basal super selective, sensitive stress response, or adrenal failure) vary depending on the duration, type, frequency, and intensity of stressors [111]. Increases in glucocorticoid levels have been found to relate to the degree of dementia in elderly people and AD patients; glucocorticoid exposure can trigger incorrect processing of amyloid precursor protein (APP) and reduce the A*β* clearance rate, thereby inducing cognitive dysfunction [112]. Chronic stress and high glucocorticoid levels also promote p-Tau accumulation [112]. Autophagy reduces misfolded proteins and polymers such as tau polymers [113], and interruption of autophagy may lead to the accumulation of protein polymers in neurodegenerative diseases. Both chronic stress and high glucocorticoid levels inhibit autophagy [114], thus accelerating the pathological process of AD. In a CUMS plus AD mouse model [115], stress exposure was found to worsen cognitive impairment induced by AD and to be closely related to metabolic disorders of sphingolipids, ketones, and amino acids in the hippocampus.

Stress, oxidative stress, and AD have an important relationship. CRS, sleep deprivation, and social isolation lead to the increase of lipid peroxidation, protein carbonyls, and nitrite in the brain [33, 42, 71, 116]; increased oxidative stress is closely related to A*β* accumulation and NFT pathology [5]; the existing A*β* induces the formation of ROS [5, 117], which can cause lipid peroxidation and protein oxidation, finally forming a vicious circle. Stress often leads to high levels of CORT, which can promote level of oxidative stress [71], while the increases in lipid peroxidation products after RS were reduced by a pharmacologic block of CORT release [72], implying that the HPA axis may play a role in stress-induced oxidative stress and increases of A*β* and NFT.

In summary, stress can mediate the occurrence of AD and aggravate cognitive impairments through various signaling pathways. Thus, it is of significance to pay attention to the influence of stress in AD patients.

### 4.2. Stress and Stroke

During the rehabilitation process, stroke patients often encounter serious challenges in movement, communication, and cognition that make it difficult to perform routine activities and tasks [118]. These challenges may affect other areas of stroke patients' lives, including their economic status and professional ability. Therefore, stroke patients face high levels of stress during the recovery process [119].

Neurovascular unit (NVU) is a multicellular complex with interdependent structures including endothelial cells, pericytes, astrocytes, microglia, neurons, and basement membrane [120, 121]. Following stroke, the level of NVU recovery is closely related to the degree of stroke recovery [122]. It has been found that [123] CRS can reduce the expression of vascular marker type IV collagen and vascular growth factors like VEGF, Ang-1, and Ang-2 following experimental stroke using the photothrombotic method. Moreover, the number of astrocytes and microglia and the expression of GFAP, Iba-1, and the neuronal marker NeuN around the infarct area in mice exposed to stroke plus CRS also reduced significantly [123]. Previous studies have shown that vascular regeneration and increases in astrocytes and microglia after stroke are beneficial to recovery [122, 124], suggesting that CRS has a negative regulatory effect on the recovery of the NVU after stroke.

It is well known that Iba-1 plays an important role in the structural reorganization of microglia, while CD11b plays a key role in the migration of microglia through the extracellular matrix [125]. When stress and stroke coexist [126], chronic stress can significantly inhibit the expression of Iba-1 and CD11b but has no effect on the expression of CD86 and MHC-II. These findings suggest that stress may not affect the phagocytic ability of microglia, but does impair the ability of microglia to migrate to the injured site, reducing the ability to clear harmful substances from neurons in time and aggravating neuronal cell death [126]. CUMS plus stroke mice also show A*β* significantly increased oligomers in the ipsilateral thalamus, which may be related to the loss of neurons [127]. Moreover, A*β* oligomers can trigger pathological activation of microglia and astrocytes by increasing the expression of peroxides and proinflammatory factors, thereby promoting neuronal cell death [128].

Oxidative stress plays an important role in stroke and its complications; amongst the most common psychiatric complications of stroke, poststroke depression (PSD) occurs during the first year after stroke onset in approximately 33% of patients [129], which may delay recovery of stroke [130]. Oxidative stress, which occurs during cerebral ischemia and the following reperfusion, is implicated in the pathogenesis of PSD [131]. PSD mice showed a decrease of GSH level and SOD activity; thus, antioxidant therapy is effective [131, 132]. However, whether stress causes or aggravates stroke injury and PSD through oxidative stress needs further research.

At present, it seems that both pre- and poststroke stress can affect stroke outcomes [133]. One study found that rats that experienced chronic stress exposure before ischemic hippocampal stroke showed higher levels of CORT, more severe brain lesions, and obvious spatial memory deficits in the ziggurat task compared to rats with ischemic stroke only [134]. However, simply making CORT elevated by injection before ischemic hippocampal stroke was insufficient to produce severe hippocampal defects relative to stroke.

Earlier studies have proposed several mechanisms involved in the expansion of brain lesions caused by stress [135]. For example, stress can lead to glutamate release, promote excitotoxicity, induce hyperglycemia [135], and inhibit the expression of antiapoptosis [136] and neurotrophic factors [137]. Autophagy is a self-supporting cellular catalytic pathway that plays an important internal role in the regulation of cell survival [138]. Previous studies have shown that autophagy, oxidative stress, and ERS are closely related [139, 140]. However, it is unknown whether stress affects ischemic stroke through autophagy; the effect of stress on stroke through oxidative stress is also vague. Therefore, further research is needed to understand the role of autophagy and oxidative stress in ischemic stroke and to study the stress level of stroke patients during the chronic recovery stage.

In a mouse model of hemorrhagic stroke established by intracerebroventricular injection of type IV collagenase [141]; CRS aggravated neurological deficits and atrophy of the hemorrhagic basal ganglia. Further research [142] showed that NVU remodeling around the hemorrhage area was delayed after CRS, which may be associated with upregulated MMP-9 and decreased tight junction- (TJ-) related proteins [142]. Moreover, after hemorrhagic stroke, CRS overactivated ERS and induced apoptosis [142].

Overall, the above studies have shown that stress can delay recovery after hemorrhagic stroke by inhibiting endogenous neuroprotective pathways and overactivating oxidative stress.

### 4.3. Stress and TBI

TBI is the result of a strong impact on the head and often coexists with mental conditions like cognitive deficits, depression, and PTSD [143]. As stress is a common factor leading to mental diseases; attention should be paid to the effect of stress on TBI. Clinical findings suggest that patients suffering from mild and moderate TBI frequently experience severe and persistent stressful events or environmental stimuli during the acute, subacute, or recovery stage [144]. As a result, such TBI patients often manifest more severe motor dysfunctions, cognitive deficits, and communication difficulties [142].

Exposure to an innate stressful stimulus before mild or moderate TBI has been shown to aggravate damage to spatial and learning acquisition and hippocampal synaptic plasticity in rats [145]. RS after moderate TBI [146] has been shown to significantly exacerbate neurological deficits, brain lesion volume, and BBB leakage via excessive activation of ER stress-mediated neurodegeneration, apoptosis, and autophagy. Further research [145] has found that stress decreases the expression of monocyte chemoattractant protein-1 (MCP-1) and macrophage inflammatory protein 1*α* (MIP-1*α*) in the cortex and increases the expression of TNF-*α* and IL-6 in the hippocampus after TBI. These findings suggest that more severe cognitive deficits in rodents after TBI plus stress may result from deterioration of the posttraumatic inflammatory process.

In the compound model of CUMS and repeated mild TBI (r-mTBI, mice experience stress first and then TBI), stress was found to reduce the increase in hippocampal pro-BDNF induced by r-mTBI [9]. Previous studies have also reported that RS after TBI decreases BDNF in rat hippocampus [144] and that BDNF levels increase after TBI to provide neuroprotection [147]. These findings indicate that stress before and after injury may hinder recovery from TBI by reducing BDNF levels.

This is also of great significance to the relationship between oxidative stress, stress, and TBI to be discussed. Many studies have found that oxidative stress is involved in the process of brain injury after TBI [148–153], and oxidative stress and neuroinflammatory sequelae of TBI contribute to posttraumatic epileptogenesis [154]. Whether stress before or after TBI can aggravate damage or cause complications through oxidative stress is unclear, but combined with the existing information, we can infer that stress may aggravate the damage caused by TBI by promoting oxidative stress, mainly based on stress-induced reduction of protective BDNF increase in the hippocampus. While under normal conditions, low level of BDNF caused by stress can prevent Nrf2 translocation and then result in sustained oxidative stress in the hippocampus [47].

Stress may also promote the occurrence of PTSD after TBI. According to previous studies [155, 156], TBI is closely related to PTSD and TBI patients show a higher PTSD rate [157]. RS after TBI consistently increases GR [144], which mediates inhibition of the HPA axis response [158]. Preexposure to stress can also aggravate damage to the HPA axis activity after mild to moderate TBI [145]. As PTSD is generally considered to relate to inhibition and enhancement of the HPA axis [159, 160], stress before or after TBI may contribute to the occurrence of PTSD.

Notably, the effect of stress on TBI is not always negative. In contrast to the above findings [161], unpredictable stress during adolescence may protect against adult TBI-induced affective and cognitive deficits [162]. Why does stress have seemingly contradictory results in TBI? This may be due to differences in stress models and the heterogeneity of TBI models. Therefore, more research is warranted to clarify the specific mechanisms that are beneficial vs. harmful to the recovery of neurological dysfunction after TBI. Variation in age and gender may also influence the neurological links between stress and TBI and require further research.

### 4.4. Stress and Epilepsy

Epilepsy is one of the most common brain diseases, with at least 50 million people worldwide suffering from epilepsy [163]. As one of the recognized causes of epilepsy, stress is expected to have a certain impact on epilepsy [164]. Manouze et al. [165] found that social isolation can aggravate the severity of epilepsy in pilocarpine-induced epileptic rats and increase seizure frequency 16-fold. In the pentylenetetrazole- (PTZ-) induced epilepsy mouse model, the latency of tonic and clonic seizures was significantly reduced and mortality was higher with RS [166]. The increase in susceptibility to epileptic seizures caused by repetitive RS may also depend on activation of hippocampal ERS, as the ERS inhibitor tauroursodeoxycholic acid (TUDCA) has been shown to reduce oxidative stress and neuron loss in the stress combined with epilepsy model [166]. Maryam et al. [167] found that exposure to stress during pregnancy changed the susceptibility of offspring to PTZ-induced seizures during a critical period of postnatal life, suggesting a cross-generational impact of stress on epilepsy. These findings indicate that stress reduces the latency of epilepsy and aggravates the severity of seizures.

## 5. Conclusion and Future Directions

According to existing studies, stress has a negative impact on brain regions through various pathways, including excessive activation of ERS and the HPA axis and oxidative stress, imbalance of E/I neurons, and abnormal changes in glia. Stress leads to neuron and astrocyte damage through ERS; although the specific mechanism remains unclear, inhibition of ERS may be a way to improve the negative effects of stress on the brain. Oxidative stress is an important way for stress to affect normal brain areas, disease development, and prognosis. Stress often aggravates oxidative stress, reduces brain antioxidant capacity, and causes emotional disorders and behavioral defects. Thus, antioxidant drugs can significantly reduce oxidative stress caused by stress and significantly improve brain injuries and diseases. In addition, magnetic treatment can partially reduce oxidative stress caused by stress [168], which may be a potential treatment.

The effect of stress on neuron plasticity is worth attention, as stress results in imbalance of E/I neurons and can lead to anxiety and depression in adult and/or newborn rodents. However, some researchers have found that mild stress may increase social behavior and reduce anxiety, indicating that “proper” stress may lead to positive effects on neuron plasticity. As is well known, neurons have high plasticity during brain development (usually childhood). Stress may lead to anxiety, depression, or other mental disorders in children or affect children's neuron plasticity in a way that increases the risk of emotional disorders in adulthood. Further research on this topic is urgently needed.

The effects of stress on brain injuries or diseases are usually considered negative. Stress aggravates cognitive deficits and lesion volume caused by stroke, TBI, and AD. The mechanisms through which stress affects brain diseases are very complex. It is known that ERS participates in the influence of stress, stroke, TBI, and epilepsy on various brain regions and may thus be a key therapeutic target. Stress can damage the BBB in stroke and TBI and even the amygdala in patients without diseases, which may be another key therapeutic point. In contrast, mild stress may ease cognitive deficits caused by TBI. Moreover, the effect of stress on glial cells is undetermined; stress activates microglia in the PFC and amygdala, but inhibits astrocytes and microglia in stroke. Therefore, the effect of stress on brain diseases and the related mechanisms need to be further studied.

Current studies have mainly revealed the effects and related molecular mechanisms of stress after brain injuries and diseases. It should be noted that most of the current research on stress is based on animal models. Although animals can represent human beings to a large extent, they are still quite different from human beings. Therefore, in the future, we need to develop research models that are closer to human beings, such as organ culture, to verify that the existing research findings apply to human beings in a state of stress.

In the future, we need to study the molecular mechanisms of stress on key brain regions and identify other possible mechanisms of stress-induced brain damage and neurological deficits. Such research will determine potential therapeutic targets and psychological interventions for patients with brain injuries and diseases who experience severe and persistent stressful events.

## Figures and Tables

**Table 1:** Various experimental stress models and their significance.

Stress model	Implementation method	Significance	Reference
Electric foot shock	Inevitable foot shock, transmitted through stainless steel mesh	Deepens fear memory Suppresses hippocampus cell proliferation Causes depression, anxiety, and PTSD	[12, 13, 169]

RS	Fixed on wood or restrained in EP pipe	Induces anxiety and depression-like behaviors	[14–17]

PPS	Exposure to predators or predator odors	High anxiety, impaired learning, and memory An excellent model to study PTSD	[18–20]

Social failure	Exposed to different resident invaders for several days	One of the most extensive animal models of depression	[29–32, 170]

CUMS	Weeks or even months of exposure to various mild stressors	One of the most widely validated and realistic depression models Closely related to human life and the natural conditions of MDD	[7, 8, 15]

MS	Separated from the mother and nest for 3 hours or more per day during the first 2 weeks after birth	Induces anxiety and depression-like behaviors in adulthood Gender differences Reduces antioxidant enzyme activity and increases lipid peroxidation in male rats May have a positive impact on anxiety behaviors	[23–27]

Social isolation	Weeks of social isolation	A type of ELS model that can simulate anxiety and depression Increases lipid peroxidation	[29–32, 34, 170]

Note: RS: restraint stress; PPS: predator-based psychosocial stress; CUMS: chronic unpredictable mild stress; MS: maternal separation; ELS: early life stress; MDD: major depressive disorder: PTSD: posttraumatic stress disorder.

**Table 2:** Neurological links between stress and key brain regions.

Brain region	Stress model	Neurological dysfunction and mechanism	Reference
Hippocampus	CUMS, CRS	Spatial memory deficits, cognitive impairments, and affective disorder ERS-mediated apoptosis of hippocampal neurons↑, caspase-3↑, Bax/Bcl-2↑ Protein/lipid peroxidation↑ and SOD↓ PI3K/AKT/mTOR-mediated BDNF/TrkB↓ NF-*κ*B↑, IL-1*β*↑, IL-6↑, and TNF-*α*↑ CA3 apical dendritic atrophy	[3, 37–40, 47, 57–59]

Hypothalamus	Acute stress, PPS, RS	Cognitive and behavioral deficits Overactivation of HPA axis nNOS mRNA↑ and SOD↓ Bidirectional changes in synaptic plasticity of PVN neuroendocrine cells Dopamine↓, dyshomeostasis of ECS Behavioral and cognitive deficits, even psychiatric disorders	[43, 60–67, 171]

Amygdala	CRS, social isolation stress, RS	Social anxiety, cognitive dysfunctions, and neurodegenerative disorders Imbalance of E/I neurons Activation of ERS of neurons and astrocytes Astrocyte apoptosis↑ Persistent oxidative status ATP↓, GR, CHOP, caspase-12↑ Overactivation of microglia ERS-mediated GABAergic BBB damage	[4, 76–80]

PFC	CUMS, ELS	Emotional disorders such as depression and anxiety E/I imbalance of neurons, neuropeptides↓ ERK-CREB-BDNF signal pathway↓ MDA↑, GSH↓, SOD↓, and BDNF↓	[55, 83–86, 90, 91]

Note: PFC: prefrontal cortex; CRS: chronic restraint stress; RS: restraint stress; PPS: predator-based psychosocial stress; CUMS: chronic unpredictable mild stress; MS: maternal separation; ELS: early life stress; GABA: *γ*-aminobutyric acid; BBB: blood-brain barrier: ERS: endoplasmic reticulum stress; ECS: endocannabinoid system; HPA: hypothalamus-pituitary-adrenal axis; E/I: excitatory/inhibitory; ↑: upregulated; ↓: downregulated.

**Table 3:** Neurobiological links between stress and brain injuries/diseases.

Compound stress model	Cognitive dysfunction and mechanism	Potential therapeutic target	Reference
Stress+AD	Stress aggravates cognitive impairments and neurodegeneration (1) Inhibition of AQP4 (2) A*β* and p-Tau accumulation↑ (3) A*β* clearance rate↓ (4) Increased A*β* production (5) ROS↑ (6) Metabolic disorders	Glucocorticoid inhibitor HPA axis inhibitor Autophagy activator AQP4 activator Antioxidant drugs	[5, 105, 109, 110, 112, 114, 115, 117]

Stress+Stroke	Brain lesions, cognitive deficits, and neurodegeneration↑ (1) Prevents angiogenesis (2) Overactivation of ERS and inhibition of astrocytes, microglia (3) ROS, neuroinflammation, and glutamate↑ (4) Glucose utilization, TJ-related protein degradation↑	Vasoactive drugs Anti-inflammatory drug Antioxidant drugs	[122, 123, 125–128, 131, 132, 141]

Stress+TBI	Cognitive impairment, lesion volume↑ (1) MCP-1, MIP-1*α*, TNF-*α*, and IL-6↑ (2) BDNF generation↓ (3) Activates ER stress-mediated neurodegeneration (4) Disorders of HPA axis	Anti-inflammatory drug BDNF ERS inhibitor, such as salubrinal Antioxidant drugs	[9, 142, 144–146, 165, 172, 173]

Stress+Epilepsy	Increases susceptibility to epilepsy Hippocampal ERS Oxidative stress↑	ERS inhibitor, such as TUDCA	[67, 79, 165–167, 174]

Note: AD: Alzheimer's disease; TBI: traumatic brain injury; ERS: endoplasmic reticulum stress; TJ: tight junction; BDNF: brain-derived neurotrophic factor; ROS: reactive oxygen species; HPA: hypothalamus-pituitary-adrenal; ↑: upregulated; ↓: downregulated.

## Data Availability

Data are available on request through the authors.

## Abbreviations

ACTH: Adrenocorticotropic hormone
AD: Alzheimer's disease
AQP4: Aquaporin 4
BBB: Blood-brain barrier
BDNF: Brain-derived neurotrophic factor
CRH: Corticotropin-releasing hormone
CUMS: Chronic unpredictable mild stress
DEX: Dexamethasone
ELS: Early life stress
ERS: Endoplasmic reticulum stress
GRP78: Glucose-regulated protein 78
GSH: L-Glutathione
HPA: Hypothalamus-pituitary-adrenal
MCP-1: Chemoattractant protein-1
MDA: Malondialdehyde
NFT: Nerve fiber tangles
Nrf2: Nuclear factor erythroid 2-related factor 2
NVU: Neurovascular unit
PFC: Prefrontal cortex
PTSD: Posttraumatic stress disorder
PTZ: Pentylenetetrazole
ROS: Reactive oxygen species
RS: Restraint stress
SOD: Superoxide dismutase
TBI: Traumatic brain injury
TNF-*α*: Tumor necrosis factor-*α*
TUDCA: Tauroursodeoxycholic acid.
